# Decreased rotational flow and circumferential wall shear stress as early markers of descending aorta dilation in Marfan syndrome: a 4D flow CMR study

**DOI:** 10.1186/s12968-019-0572-1

**Published:** 2019-10-14

**Authors:** A. Guala, G. Teixido-Tura, L. Dux-Santoy, C. Granato, A. Ruiz-Muñoz, F. Valente, L. Galian-Gay, L. Gutiérrez, T. González-Alujas, K. M. Johnson, O. Wieben, A. Sao Avilés, A. Evangelista, J. Rodriguez-Palomares

**Affiliations:** 1 Hospital Universitari Vall d’Hebron, Department of Cardiology. CIBER-CV. Vall d’Hebron Institut de Recerca (VHIR), Universitat Autònoma de Barcelona, Paseo Vall d’Hebron 119-129, 08035 Barcelona, Spain; 2 Departments of Medical Physics & Radiology, University of Wisconsin – Madison, Madison, WI USA

**Keywords:** Marfan syndrome, 4D flow CMR, Helical flow, Descending aorta, Aortic aneurysm, Wall shear stress (WSS)

## Abstract

**Background:**

Diseases of the descending aorta have emerged as a clinical issue in Marfan syndrome following improvements in proximal aorta surgical treatment and the consequent increase in life expectancy. Although a role for hemodynamic alterations in the etiology of descending aorta disease in Marfan patients has been suggested, whether flow characteristics may be useful as early markers remains to be determined.

**Methods:**

Seventy-five Marfan patients and 48 healthy subjects were prospectively enrolled. In- and through-plane vortexes were computed by 4D flow cardiovascular magnetic resonance (CMR) in the thoracic aorta through the quantification of in-plane rotational flow and systolic flow reversal ratio, respectively. Regional pulse wave velocity and axial and circumferential wall shear stress maps were also computed.

**Results:**

In-plane rotational flow and circumferential wall shear stress were reduced in Marfan patients in the distal ascending aorta and in proximal descending aorta, even in the 20 patients free of aortic dilation. Multivariate analysis showed reduced in-plane rotational flow to be independently related to descending aorta pulse wave velocity. Conversely, systolic flow reversal ratio and axial wall shear stress were altered in unselected Marfan patients but not in the subgroup without dilation. In multivariate regression analysis proximal descending aorta axial (*p* = 0.014) and circumferential (*p* = 0.034) wall shear stress were independently related to local diameter.

**Conclusions:**

Reduced rotational flow is present in the aorta of Marfan patients even in the absence of dilation, is related to aortic stiffness and drives abnormal circumferential wall shear stress. Axial and circumferential wall shear stress are independently related to proximal descending aorta dilation beyond clinical factors. In-plane rotational flow and circumferential wall shear stress may be considered as an early marker of descending aorta dilation in Marfan patients.

**Electronic supplementary material:**

The online version of this article (10.1186/s12968-019-0572-1) contains supplementary material, which is available to authorized users.

## Introduction

Marfan syndrome is a hereditary connective tissue disorder caused by a mutation in the FBN1 gene. The remarkable advances in diagnosis, treatment and elective aortic root replacement resulted in a great reduction in proximal aorta fatal events in Marfan patients [1, 2], prompting an impressive rise in life expectancy [3]. As a result, diseases of the descending aorta (DAo) have emerged as a clinical issue, either in the form of a primary complication of the DAo or in the follow-up of patients with previous surgical prophylactic ascending aorta replacement [1, 2, 4]. Longitudinal data in Marfan patients showed that 63% of aortic dissections involved the distal aorta, in 31% of which the involvement was exclusive [4].

Although aortic diameter has been identified as a risk marker for DAo complications [1, 2, 4], 47% of type B aortic dissections occur with DAo diameter < 27 mm [1]. As a consequence, other risk markers beyond aortic diameter are needed to better define the risk of DAo complications in these patients [4].

For this purpose, 4D flow cardiovascular magnetic resonance (CMR) studies evaluating hemodynamics and wall shear stress (WSS) have recently been conducted. Wang et al. reported reduced helical flow in the aortic root in MFS patients [5], while other studies demonstrated the role of proximal DAo vortexes in the creation of areas of low WSS localized in the proximal DAo [6–8], which were related to local dilation [6] in a region where most type B dissections occur [6]. However, several aspects have not been investigated to date. Firstly, no study made a quantitative evaluation of these flow abnormalities. Thus, previous semi-quantitative analyses were limited for differentiating a pathologic from a physiologic proximal DAo vortex [6, 9], and characterizing these vortexes in terms of direction and intensity. Moreover, no studies to date have evaluated these flow disturbances in MFS without aortic dilation, thereby limiting the possibility of assessing whether these flow characteristics may be early markers of aortic disease.

We aimed to investigate blood flow and WSS patterns by 4D flow CMR in the thoracic aorta of Marfan patients with and without aortic dilation to identify potential early markers of DAo disease.

## Methods

### Study population

Seventy-five genetically-confirmed Marfan syndrome patients were prospectively recruited from our Aortic Unit. Inclusion criteria were: age > 18 years and absence of bicuspid aortic valve, significant valve dysfunction (<grade III aortic regurgitation and stenosis) and previous heart or aortic surgery and contraindication for CMR . Furthermore, 48 healthy subjects were recruited as controls. The study was approved by the local ethics committee and written informed consent was obtained from all participants.

### Cardiovascular magnetic resonance protocol

CMR studies were performed on a clinical 1.5 T scanner (Signa, GE Healthcare, Waukesha, Wisconsin , USA). The protocol included balanced steady-state free-precession (bSSFP) cine imaging to assess aortic diameter, 2D phase contrast images of the aortic valve to evaluate aortic valve disease and a 4D phase-contrast CMR (4D flow CMR) acquisition for hemodynamics analysis. A radially-undersampled acquisition (PC VIPR) with 5-point balanced velocity encoding [10] with retrospective electrocardiogram (ECG)-gating during free-breathing was used for 4D flow imaging of the entire thoracic aorta in ≈ 10 min total scan time. Data were acquired with an eight-channel cardiac coil (HD Cardiac, GE Healthcare) using the following parameters: velocity encoding (VENC) 200 cm/s, field of view 400x400x400 mm, acquisition matrix 160x160x160, voxel size 2.5 × 2.5 × 2.5 mm. This data set was reconstructed offline according to the nominal temporal resolution (5xTR) of each patient, yielding a temporal resolution of 25.5 ± 5 ms. Data were corrected for background phase from concomitant gradients, eddy currents and trajectory errors of the 3D radial acquired k-space [10]. Brachial systolic (SBP) and diastolic (DBP) pressures were taken immediately after the CMR study.

### Hemodynamics evaluation

Patient-specific 3D geometric models of the aorta were semi-automatically reconstructed from Phase Contrast Magnetic Resonance Angiograms (PC MRA) using ITK-Snap [11] and used to mask the velocity data. PC MRA was used to identify the sinotubular junction (STJ), first and last supra-aortic vessels and location of the pulmonary artery bifurcation. The height of the pulmonary artery bifurcation served to separate proximal and distal regions of both ascending (AAo) and DAo. Aortic centerline was computed and 20 perpendicular analysis planes were identified between the STJ and end of the proximal DAo. Eight equidistant analysis planes were located in the AAo, 4 in the aortic arch and 8 in the proximal DAo, yielding average distances between analysis planes of 10 mm in the AAo, 7.2 mm in aortic arch and 7.4 mm in the proximal DAo.

**Fig. 1:**
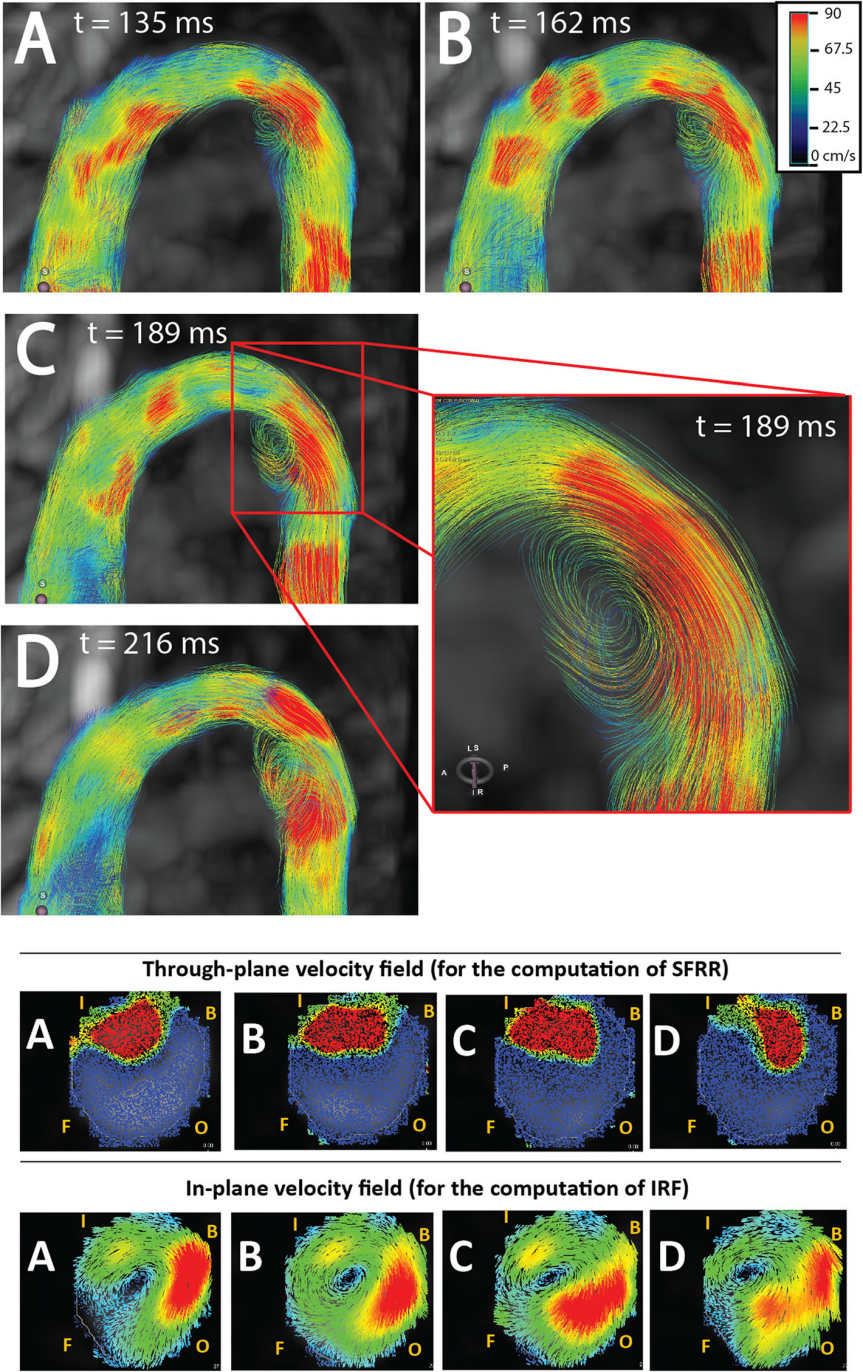
Flow field visualization. Top: visualization of the velocity field as a series of sagittal images showing flow streamlines at four successive systolic time frames (from **a** to **d**, 27 ms time step) of a representative Marfan patient with dilation of the proximal descending aorta (DAo). Streamline visualization was obtained with CVI42, Circle Cardiovascular Imaging Inc., Calgary, Canada. Bottom: through-plane (red, backward with |velocity| > 10 cm/s; blue, forward with |velocity| > 10 cm/s) and in-plane (red, higher velocity, same velocity scale as top boxes.) velocity components of a proximal DAo section of the same patient. B = back, F = front, I = inner, IRV = in-plane rotational flow, O = outer,SFRR = systolic flow reversal ratio

Hemodynamics characterization was made in each plane using custom-designed Matlab (Mathworks, Natick, Massachusetts,USA) code. In-plane rotational flow (IRF), also called circumferential circulation [12], a widely-used marker, was quantified using circulation, a parameter used in fluid dynamics to quantify flow rotation. IRF is a surrogate marker of helical flow that quantifies its circumferential part by isolating the rotational component of the velocity field residing in the plane [13]. In-plane rotational flow was computed at peak systole, averaging through one time frame before and two frames after peak systole to mitigate noise. Systolic flow reversal ratio (SFRR) was calculated as the ratio of forward to backward through-plane systolic volumes [14]. This parameter, also known as systolic backward flow, offers quantification of the strength of vortexes rotating around an axis perpendicular to the centerline. Figure 1 permits visualization of the differences between in-plane (IRF) and through-plane (SFRR) flow rotation patterns in a representative Marfan patient with proximal DAo dilation. The maximum through-plane velocity at the STJ was also extracted.

Axial and circumferential WSS were computed as previously described [15, 16]. In order to compute population-averaged WSS maps, axial and circumferential WSS were averaged at 8 regions around the lumen contour of each cross-sectional plane. Data were aligned for all patients using the inner aortic curvature as a reference. Axial and circumferential WSS were averaged over meaningful regions to test whether they are independently related to proximal DAo diameter. Axial WSS was averaged in the left/left-inner region of the proximal DAo (planes 14 and 15, where dilation is most likely to be present), while circumferential WSS was averaged on the circular section 14. Thus, averaging was made in the region where the peak curvature and most flow abnormalities are seen [7].

### Aortic mechanical properties

Regional aortic pulse wave velocity was computed separately in the ascending and descending aorta as previously described [17]. Briefly, local velocity waveforms were extracted at 100 equally-distributed analysis planes and transit time was calculated by wavelet analysis as recently proposed [18].

### Aortic diameters and definitions of dilation

The three cusp-to-cusp diameters were measured at the aortic root level at the end-diastolic frame and the maximum was considered for analysis. Aortic root dilation was considered when z-score, based on age, body surface area (BSA) and sex as reported by Devereux et al. [19], was > 2. Diameter of the DAo at pulmonary artery level was extracted from PC MRA. DAo dilation was defined as a diameter > 90th percentile of the sex-, age- and BSA-matched population published by Rogers et al. [20]. The non-dilated MFS subgroup included patients not presenting with dilation of either the aortic root or the DAo.

### Aortic valve disease

PC images of the aortic valve were used to evaluate aortic valve disease. Aortic valve stenosis was evaluated by extracting the maximum velocity and aortic valve regurgitation via regurgitant fraction.

### Statistical analysis

Continuous demographic variables were expressed as mean ± standard deviation if they presented a normal distribution, and as median [1st-3rd] quartiles otherwise. Categorical variables were presented as frequency (percentage). The Kolmogorov-Smirnov test was used to evaluate distribution normality. Differences between groups for continuous parameters were assessed by Student’s t-test if normally distributed, and Mann-Whitney U test otherwise. Chi-square test was used for categorical variables. Multivariate linear or logistic regression analyses with a backward selection procedure and multicollinearity test were used to identify statistically-significant associations. Independent variables entered the model if *p* < 0.15 in univariate analyses. A two-tailed *p* value < 0.05 was considered statistically significant. SPSS 21.0 (Statistical Package for the Social Sciences, International Business Machines, Inc., Armonk, New York, USA) was used for the analysis.

## Results

### Demographic characteristics

Demographic characteristics, aortic diameters and regional aortic stiffness in healthy subjects and Marfan patients with and without aortic dilation are shown in Table 1.

**Table 1:** Demographics and clinical data

	Healthy Subjects	Marfan patients					
		ALL		Non-dilated Marfan		Dilated Marfan	
N	48	75	*p*-values	20	*p*-values	55	*p*-values
age [years]	39 ± 12	37 ± 13	0.327	34 ± 10	0.104	38 ± 14	0.611
Sex [N, (%) men]	31 (65)	42 (56)	0.026	5 (25)	0.002	37 (67)	0.165
Height [cm]	172 ± 8	180 ± 11	< 0.001	177 ± 9	0.024	182 ± 11	< 0.001
BSA [m^2^]	1.84 ± 0.16	1.91 ± 0.22	0.052	1.85 ± 0.22	0.931	1.95 ± 0.22	0.011
SBP [mmHg]	126 ± 18	128 ± 17	0.523	130 ± 15	0.381	128 ± 18	0.704
DBP [mmHg]	70 ± 12	74 ± 12	0.069	76 ± 11	0.051	74 ± 12	0.176
AoV regurgitant fraction [%]	1 ± 1	2 ± 5	0.087	1 ± 1	0.593	3 ± 5	0.035
AoV peak velocity [m/s]	1.2 ± 0.2	1.1 ± 0.2	0.067	1.1 ± 0.2	0.057	1.1 ± 0.3	0.122
Aortic root diameter [mm]	30.0 ± 4.0	38.6 ± 5.0	< 0.001	34.0 ± 3.1	< 0.001	40.3 ± 4.7	< 0.001
Aortic root z-score	−0.73 ± 1.06	2.32 ± 1.99	< 0.001	0.69 ± 0.94	< 0.001	2.88 ± 1.79	< 0.001
AAo diameter [mm]	30.2 ± 4.1	35.2 ± 6.4	< 0.001	30.1 ± 6.1	0.958	37.1 ± 5.8	< 0.001
Proximal DAo diameter [mm]	23.2 ± 2.9	25.1 ± 4.4	0.009	22.2 ± 2.1	0.174	26.2 ± 4.5	< 0.001
AAo PWV [m/s]	5.3 ± 1.9	7.3 ± 2.8	< 0.001	7.1 ± 2.2	0.002	7.3 ± 3.0	< 0.001
DAo PWV [m/s]	7.2 ± 2.2	10.8 ± 4.5	< 0.001	10.2 ± 4.0	< 0.001	11.1 ± 4.6	< 0.001

Demographic characteristics, aortic diameters and ascending and descending pulse wave velocities (PWV) of healthy volunteers (HV), Marfan patients (ALL MFS) and the subset of Marfan patients with neither aortic root nor descending aorta dilation (Non-dilated Marfan). SBP, DBP: systolic and diastolic blood pressure, respectively. AAo represents ascending and DAo descending aorta. Data are presented as mean ± SD or number (percentage). *P*-values report the comparison with healthy controls

As expected, Marfan patients had larger aortic root and AAo and DAo diameters. Twenty Marfan patients free of dilation of the aortic root and the proximal DAo were grouped as non-dilated Marfan subjects. Non-dilated Marfan had larger aortic root diameter compared with healthy subjects; however, aortic root z-score was low and within normal range. DBP was slightly but not significantly higher in Marfan compared to healthy subjects . Mean aortic valve regurgitant fraction and maximum velocity were slightly higher and lower, respectively, in Marfan compared to healthy subjects . However, differences were clinically insignificant since the values fell within normal range. As previously reported [17, 21, 22], Marfarn patients presented increased AAo and DAo stiffness (measured here as an increase in pulse wave velocity) compared with healthy subjects, even in the absence of dilation.

### Hemodynamics

**Fig. 2:**
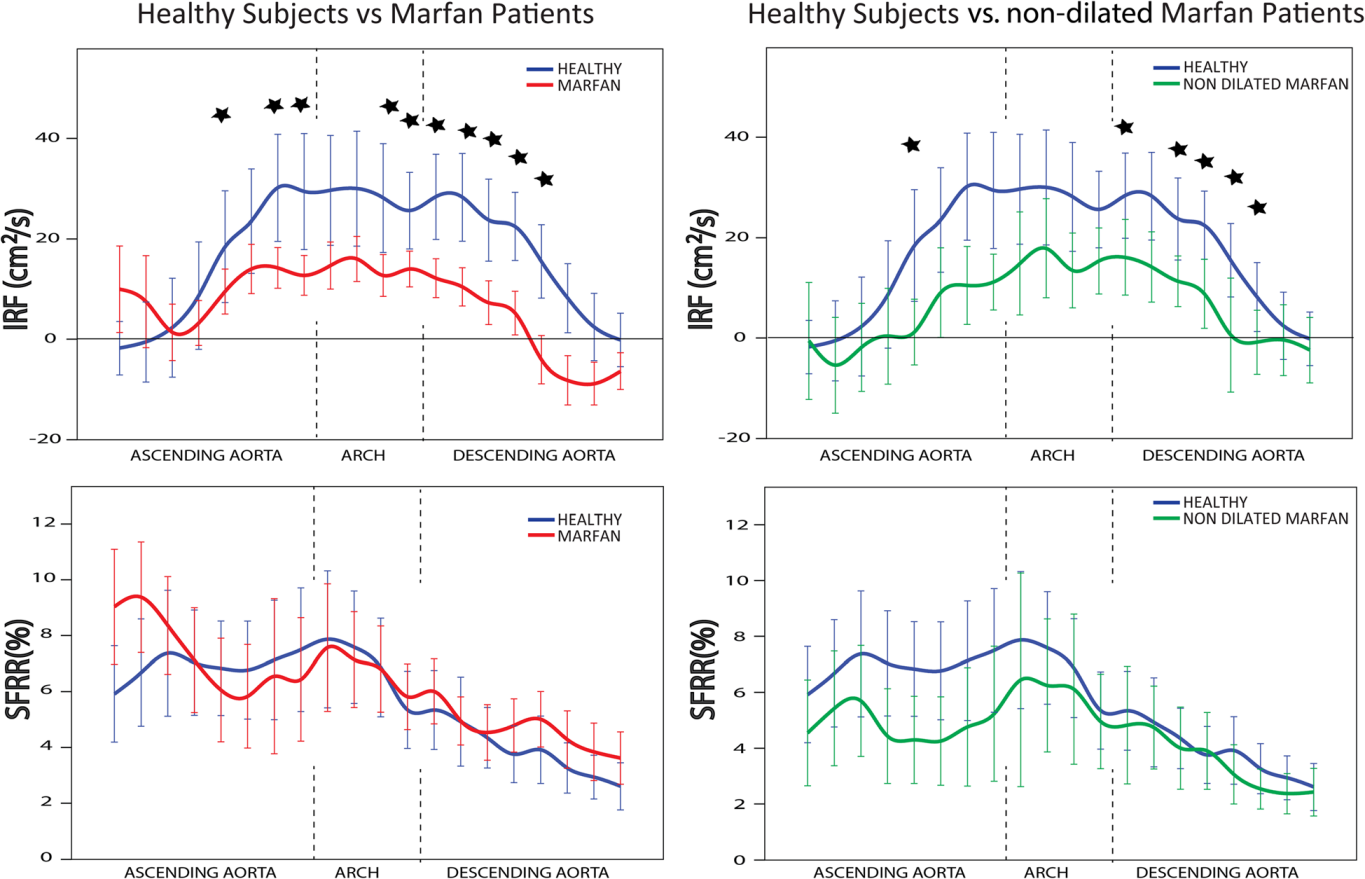
Rotational flow characteristics. In-plane rotational flow (IRF) (top) and systolic flow reversal ratio (SFRR) (bottom) at the 20 analysis planes. Blue lines and box plots present data for healthy subjects, red regards all Marfan syndrome patients (left) while green reports data from non-dilated Marfan syndrome patients (right).* Statistically-significant (*p* < 0.05) difference between the groups after multivariate corrections

IRF, which is the in-plane projection of helical flow, was substantially lower in Marfan patients at the distal AAo, aortic arch and proximal DAo (see top-left panel of Fig. 2 and Additional file 1: Table S1). Differences were statistically-significant in most planes between distal AAo and proximal DAo, even after the inclusion in multivariate analysis of sex, height, BSA, DBP, aortic valve regurgitant fraction and maximum velocity as independent variables. Interestingly, Marfan transitioned from clockwise to counter-clockwise rotation (i.e. from positive to negative IRF) in the middle of the proximal DAo. Conversely, in healthy subjects , IRF was progressively reduced when moving distally, without presenting inversion of rotation direction, at least before pulmonary bifurcation level. SFRR, also called systolic backward flow being a measurement of backward systolic flow and thus systolic through-plane vorticity, tended to be higher in Marfan compared to healthy subjects in the proximal AAo and DAo, but not in the aortic arch (see bottom-left panel of Fig. 2). However, on multivariate analysis, none of these tendencies reached statistical significance.

Interestingly, even the subset of 20 Marfan patients without aortic dilation presented a markedly lower IRF at the distal AAo and proximal DAo compared to healthy subjects (see top-right panel of Fig. 2). These differences were statistically-significant on multivariate analysis after inclusion of sex, age, height, DBP, aortic root diameter and aortic valve regurgitant fraction and maximum velocity as independent variables. This means that a reduction in in-plane rotational flow is present in Marfan without clinically-significant aortic dilation. Conversely, SFRR was lower in non-dilated Marfan patients, thereby suggesting that the increase in such a flow alteration may partially result from dilation.

In univariate analysis in all Marfan patients, average IRF over the ascending aorta, aortic arch and proximal descending aorta was related to BSA (*R* = 0.28), maximum velocity at the sinotubular junction (*R* = 0.270) and DAo PWV (*R* = -0.30) but not with age, sex, systolic and diastolic blood pressure, aortic root z-score and AAo PWV (see Table 2). In multivariate analysis corrected for BSA and maximum velocity at the sinotubular junction, average IRF was independently related to DAo PWV (*p* = 0.041).

**Table 2:** univariate and multivariate linear regression analysis for thoracic aorta mean IRF in Marfan patients

	Univariate	Multivariate		
	*p*-value	*p*-value	B	IC
Age [years]	0.637	–	–	–
BSA [m^2^]	0.020	0.047	0.235	[−0.129;17.528]
Sex [male]	0.110	–	–	–
SBP [mmHg]	0.117	–	–	–
DBP [mmHg]	0.438	–	–	–
Aortic root z-score	0.203	–	–	–
Vmax [cm/s]	0.020	0.050	0.231	[0.000; 0.138]
AAo PWV [m/s]	0.165	–	–	–
DAo PWV [m/s]	0.013	0.041	−0.232	[−0.875; −0.019]

SBP, DBP: systolic and diastolic blood pressure, respectively. AAo represents ascending and DAo descending aorta, Vmax is the maximum through-plane velocity at the sinotubular junction and PWV means pulse wave velocity

### Axial and circumferential WSS maps

**Fig. 3:**
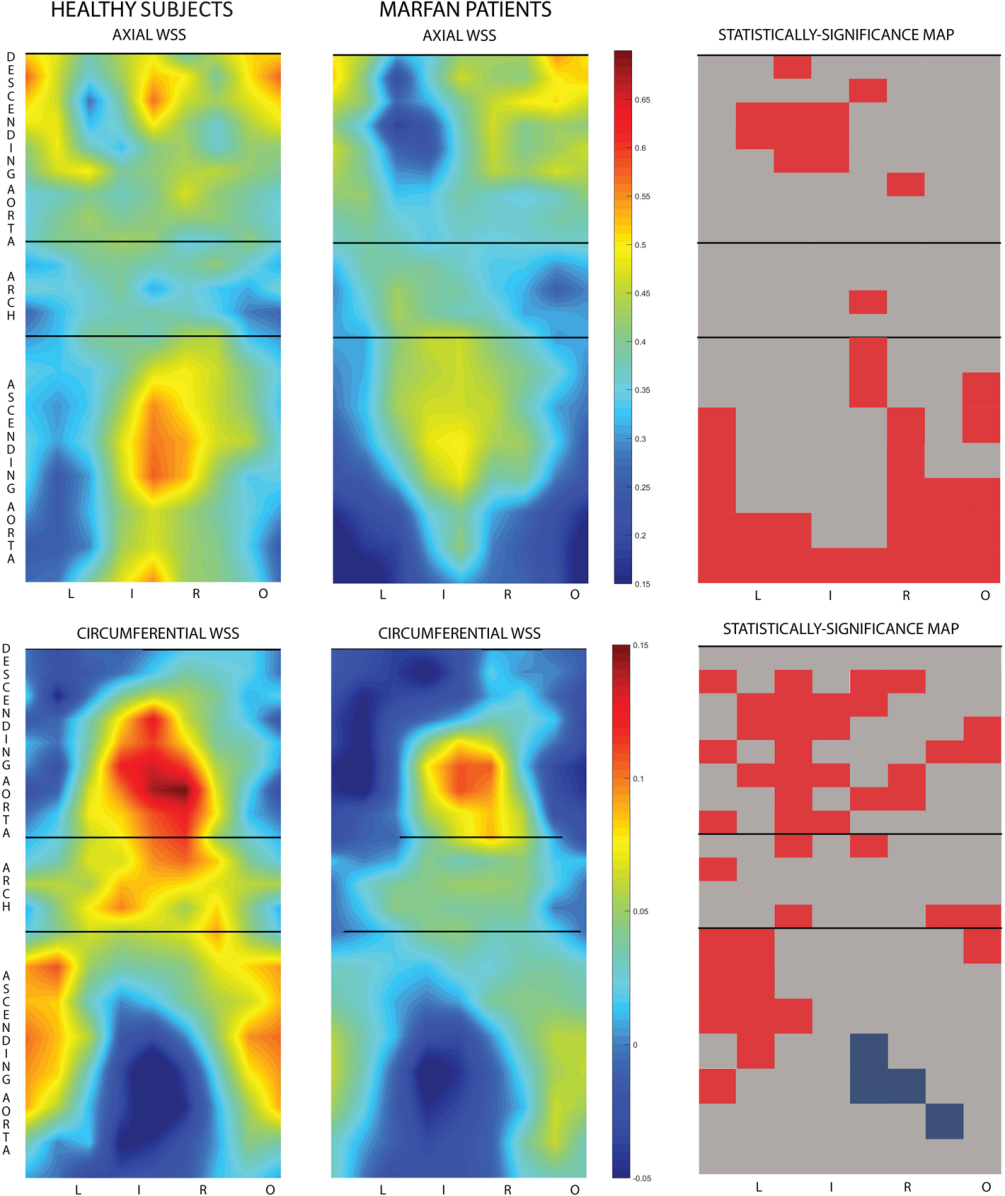
WSS maps in healthy subjects and in Marfan patients. Axial (top) and circumferential (bottom) wall shear stress (WSS) in the ascending aorta, aortic arch and proximal descending aorta in healthy subjects (left) and in Marfan patients (center). (Right) Statistically-significance differences (*p* < 0.05) maps after multivariate corrections (red = healthy subjects higher, blue = Marfan higher)

Axial WSS maps showed lower values in Marfan patients compared to healthy subjects in the proximal AAo, especially in the outer region, and in the left-inner region of the proximal DAo (see top row of Fig. 3). Circumferential WSS was reduced in Marfan patients in the left-outer regions of the distal AAo and proximal aortic arch and in the left-inner regions of the proximal DAo (see bottom row of Fig. 3). Regions of statistically-significant differences in multivariate analysis are shown in the right panel of Fig. 3.

**Fig. 4:**
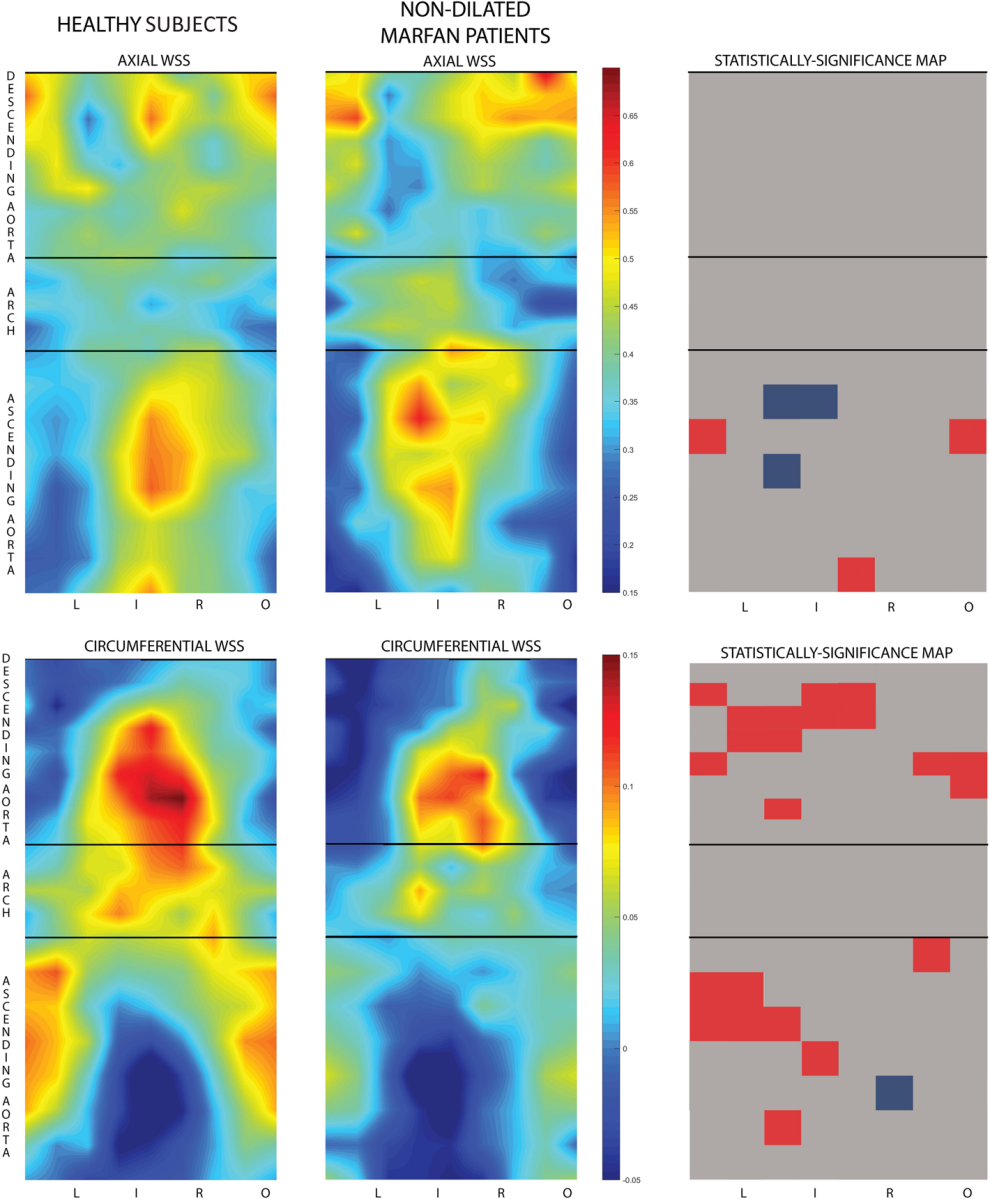
WSS maps in healthy subjects and in non-dilated Marfan patients Axial (top) and circumferential (bottom) wall shear stress (WSS) in the ascending aorta, aortic arch and proximal descending aorta in healthy subjects (left) and in Marfan patients without aortic dilation (center). (Right) Statistically-significance differences (*p* < 0.05) maps after multivariate corrections (red = healthy subjectshigher, blue = Marfan higher)

Non-dilated Marfan patients presented limited regions of altered axial or circumferential WSS in the proximal AAo compared to HV (see Fig. 4). The reduction in circumferential but not axial WSS was statistically-significant in the left/inner regions of the proximal DAo on multivariate analysis.

Mean axial WSS in the left/left-inner region in the proximal DAo was lower in MFS patients compared with healthy subjects(0.315 vs 0.429 N/m^2^, respectively, *p* = 0.010), even after multivariate adjustment (*p* = 0.018). However, though similar, the difference with healthy subjects was not statistically-significant (0.309 N/m^2^, *p* = 0.065) in non-dilated Marfan. Contour-averaged circumferential WSS in the proximal DAo was strongly related to local IRF (*R* = 0.805, *p* < 0.001) and was reduced in the overall Marfan cohort (0.021 vs. 0.065 N/m^2^, respectively. *p* < 0.001) and in the non-dilated group (0.028 N/m^2,^ *p* = 0.024) compared to healthy subjects , even after multivariate adjustments (*p* = 0.001 and *p* = 0.042, respectively).

### Multivariate determinants of proximal DAo diameter

Univariate and multivariate correlates of DAo maximum diameter are described in Table 3. In multivariate analysis, local contour-averaged circumferential WSS (*p* = 0.034) and axial WSS in the inner wall (*p* = 0.014) were significantly and independently related to maximum proximal DAo diameter after correction for age and BSA (see Table 3). Of note, the performance of the multivariate model was very good, with an adjusted R^2^ of 54%.

**Table 3:** univariate and multivariate linear regression analysis for proximal DAo maximum diameter in MFS patients

	Univariate	Multivariate		
	*p*-value	*p*-value	B	IC
Age [years]	< 0.001	< 0.001	0.583	[0.136; 0.247]
BSA [m^2^]	0.020	< 0.001	0.416	[4.815;11.326]
SBP [mmHg]	0.007	–	–	–
DBP [mmHg]	0.421	–	–	–
Aortic root diameter [mm]	0.048	–	–	–
AAo PWV [m/s]	0.421	–	–	–
DAo PWV [m/s]	0.044	–	–	–
Circ. WSS_14_	0.021	0.034	−0.182	[−37.4; −1.5]
Proximal DAo inner axial WSS	0.019	0.014	−0.218	[−9.2; −1.1]

SBP, DBP: systolic and diastolic blood pressure, respectively. AAo represents ascending and DAo descending aorta, PWV means pulse wave velocity and WSS wall shear stress

## Discussion

This study analyzed blood flow and WSS patterns as well as aortic stiffness by 4D flow CMR in the thoracic aorta of a large cohort of Marfan patients with and without aortic dilation to identify potential early markers of descending aorta disease. To our knowledge, this is the first study to quantitatively evaluate all these interrelated aspects in Marfarn patients.

Vortexes in the proximal DAo of unselected Marfan adults were found to differ from those of healthy individuals. These vortexes were characterized by a large reduction in in-plane rotational flow (IRF, the in-plane projection of helical flow) in the distal ascending aorta, aortic arch and proximal DAo and a limited increase in through-plane vortexes (SFRR) located in dilation regions. Moreover, IRF but not SFRR was impaired in Marfan patients without aortic root or DAo dilation (see Additional file 2: Video 1). Similar results were obtained for wall shear stress, the circumferential, but not axial, component of which was reduced even in non-dilated patients. A marked, positive relationship between in-plane rotational flow and circumferential WSS was observed, as expected. Indeed, viscous and inertial forces tend to render the velocity field between innermost (where most of the voxels included in IRF computation are located) and outermost layers, where WSS is calculated, uniform. This positive relationship had already been reported or at least suggested by a number of studies [23–25], which emphasized the role of helical flow-induced WSS to limit platelet activation, atherogenic lipid adhesion and energy dissipation [26, 27]. Finally, in multivariate analysis corrected for age and BSA, both local circumferential and axial WSS in the proximal DAo were significantly and independently related to local diameter.


**Additional file 2:** Representative video comparing a dilated Marfan patient (right), a non-dilated Marfan patient (center) and a healthy controls (left). (MP4 8940 kb)


Vortexes in the proximal DAo of adolescent Marfan patients were recently identified by semi-qualitative visual inspection of 4D flow CMR studies [6–8]. However, none of those studies differentiated between rotation axis directions [6–8]. Interestingly, the present study found increased systolic flow reversal ratio (SFRR) to be absent in non-dilated Marfan patients, thereby indicating that through-plane vortexes might be a consequence of local dilation. On the other hand, in-plane vortexes, as identified through the computation of in-plane rotational flow, were found to be reduced in the distal AAo and aortic arch. This concurs with a quantitative study reporting reduced helical flow in the AAo in a small population of adult Marfan patients [5]. Our data further showed the reduction in IRF to be highly related to a reduction in circumferential WSS, occur even in non-dilated Marfan patients and be independently related to aortic stiffness. To appreciate the significance of this finding, it is important to consider the physiologic role of helical flow. Indeed, it is thought to limit the separation of flow from the arterial wall when flowing through regions with sudden geometric or mechanical heterogeneity (such as bifurcations), thereby limiting energy dissipation [23–25, 28]. In other words, a coherent helical flow pattern results from evolution aimed at obtaining more efficient blood flow [24, 25, 29]. In light of this, impaired IRF, a proxy of helical flow, in Marfan patients could effectively be seen as a pathologic characteristic. Regarding the origin of impaired helical flow in Marfan patients, our data revealed an independent inverse relationship with descending aorta stiffness and not with aortic root z-score and age, thereby suggesting a role for aortic stiffness in the creation of this abnormal flow feature. Of note, reduced IRF highlights a difference with respect to bicuspid aortic valve patients who present abnormally elevated IRF [13, 15].

Interestingly, helical flow rotated counterclockwise in the middle of the proximal DAo in Marfan patients, the site of maximum diameter. Counterclockwise rotational flow has been suggested as a more severe flow alteration in bicuspid aortic valve patients [30].

WSS maps revealed axial WSS to be reduced in the dilation-affected regions. However, comparing non-dilated Marfan patients with healthy subjects showed a limited number of regions with statistically-significant differences in axial WSS. By contrast, circumferential WSS was reduced in both dilated and non-dilated Marfan patients and may thus be an early marker of disease. The reduction in axial and circumferential WSS seen in the present study supports previous findings in a much smaller Marfarn population [5].

Multivariate analysis showed circumferential and axial WSS to be independent correlates of proximal DAo diameter beyond age, BSA and regional stiffness. This result adds to previous findings [6] with respect to the vectorial nature of WSS, and further suggests that local hemodynamics may be superior to local stiffness in the etiology of DAo dilation. Despite the need for longitudinal studies to demonstrate a potential causative role, these data suggest that the WSS measurement may be important in the clinical management of Marfarn patients and deserve further longitudinal studies.

### Limitations

IRF and WSS were computed by averaging the flow field of three time frames around peak systole [15]. This method permits noise the reduction but can result in extremely rapid fluctuations being missed. Furthermore, as IRF is computed over the whole cross-section, the topology of local secondary flow structures could not be depicted. The 4D flow studies were acquired without respiratory gating. This should not imply substantial differences in the descending aorta, where respiratory motion is limited [31], especially during tidal breathing [32], as in the present investigation. The cross-sectional nature of the study implies the impossibility of investigating causal relationship between variables. The capacity of IRF and circumferential WSS to predict outcomes should thus be assessed in longitudinal studies.

## Conclusions

Impaired in-plane rotational flow and circumferential wall shear stress are present in Marfan patients regardless of aortic dilation. Reduced axial and circumferential wall shear stress in the proximal descending aorta are independently related to local diameter beyond demographics and classic clinical factors.

## Additional file

Additional file 1: Numerical results included in Fig. 2.(DOCX 27 kb)

## Data Availability

The data that support the findings of this study are available from the corresponding author upon reasonable request.

## Abbreviations

3D: Three-dimensional
4D flow CMR: Time-resolved three-dimensional phase-contrast cardiac magnetic resonance imaging
AAo: Ascending aorta
BSA: Body surface area
bSSFP: balanced steady state free precession
CMR: Cardiovascular magnetic resonance
DAo: Descending aorta
DBP: Diastolic blood pressure
ECG: Electrocardiogram
IRF: In-plane rotational flow
MRA: Magnetic resonance angiography
PC: Phase contrast
PWV: Pulse wave velocity
SBP: Systolic blood pressure
SFRR: Systolic flow reversal ratio
STJ: Sinotubular junction
VENC: Velocity encoding
WSS: Wall shear stress

## References

[1] Den Hartog AW, Franken R, Zwinderman AH (2015). The risk for type B aortic dissection in Marfan syndrome. J Am Coll Cardiol.

[2] Engelfriet PM, Boersma E, Tijssen JGP, Bouma BJ, Mulder BJM (2006). Beyond the root: dilatation of the distal aorta in Marfan’s syndrome. Heart..

[3] Silverman DI, Burton KJ, Gray J (1994). Life expectancy in the Marfan syndrome. Am J Cardiol.

[4] Mimoun L, Detaint D, Hamroun D (2011). Dissection in Marfan syndrome: the importance of the descending aorta. Eur Heart J.

[5] Wang HH, Chiu HH, Tseng WYI, Peng HH (2016). Does altered aortic flow in marfan syndrome relate to aortic root dilatation?. J Magn Reson Imaging.

[6] Geiger J, Hirtler D, Gottfried K (2017). Longitudinal evaluation of aortic hemodynamics in Marfan syndrome: new insights from a 4D flow cardiovascular magnetic resonance multi-year follow-up study. J Cardiovasc Magn Reson.

[7] Geiger J, Markl M, Herzer L (2012). Aortic flow patterns in patients with Marfan syndrome assessed by flow-sensitive four-dimensional MRI. J Magn Reson Imaging.

[8] van der Palen RLF, Barker AJ, Bollache E (2017). Altered aortic 3D hemodynamics and geometry in pediatric Marfan syndrome patients. J Cardiovasc Magn Reson.

[9] Hope TA, Markl M, Wigstrom L, Alley MT, Miller DC, Herfkens RJ (2007). Comparison of flow patterns in ascending aortic aneurysms and volunteers using four-dimensional magnetic resonance velocity mapping. J Magn Reson Imaging.

[10] Johnson KM, Lum DP, Turski PA, Block WF, Mistretta CA, Wieben O (2009). Improved 3D phase contrast MRI with off-resonance corrected dual Echo VIPR. Magn Reson Med.

[11] Yushkevich PA, Piven J, Hazlett HC (2006). User-guided 3D active contour segmentation of anatomical structures: significantly improved efficiency and reliability. Neuroimage..

[12] Hess AT, Bissell MM, Glaze SJ, Pitcher A, Myerson SG, Neubauer S (2013). Evaluation of circulation, Γ, as a quantifying metric in 4D flow MRI. J Cardiovasc Magn Reson.

[13] Dux-Santoy L, Guala A, Teixidó-Turà G, Ruiz-Muñoz A, Maldonado G, Villalva N (2019). Increased rotational flow in the proximal aortic arch is associated with its dilation in bicuspid aortic valve disease. Eur Hear J - Cardiovasc Imaging.

[14] Bensalah MZ, Bollache E, Kachenoura N (2014). Geometry is a major determinant of flow reversal in proximal aorta. Am J Physiol Hear Circ Physiol.

[15] Rodríguez-Palomares JF, Dux-Santoy L, Guala A, et al. Aortic flow patterns and wall shear stress maps by 4D-flow MRI in the assessment of aortic dilatation in bicuspid aortic valve. J Cardiovasc Magn Reson. 2018;20(28). 10.1186/s12968-018-0451-1. 10.1186/s12968-018-0451-1 PMC5918697 29695249

[16] Guala A, Rodriguez-Palomares J, Galian-Gay L (2019). Partial aortic valve leaflet fusion is related to deleterious alteration of proximal aorta hemodynamics. Circulation..

[17] Guala A, Rodríguez-Palomares JF, Dux-Santoy L (2019). Influence of aortic dilation on the regional aortic stiffness of bicuspid aortic valve assessed by 4-dimensional flow cardiac magnetic resonance. JACC Cardiovasc Imaging.

[18] Bargiotas I, Mousseaux E, Yu W (2015). Estimation of aortic pulse wave transit time in cardiovascular magnetic resonance using complex wavelet cross-spectrum analysis. J Cardiovasc Magn Reson.

[19] Devereux RB, de Simone G, Arnett DK (2012). Normal limits in relation to age, body size and gender of two-dimensional echocardiographic aortic root dimensions in persons ≥15 years of age. Am J Cardiol.

[20] Rogers IS, Massaro JM, Truong Q (2013). Distribution, determinants, and normal reference values of thoracic and abdominal aortic diameters by computed tomography (from the Framingham heart study). Am J Cardiol.

[21] Teixido-Tura G, Redheuil A, Rodríguez-Palomares JF (2014). Aortic biomechanics by magnetic resonance: early markers of aortic disease in Marfan syndrome regardless of aortic dilatation?. Int J Cardiol.

[22] Guala Andrea, Teixidó-Tura Gisela, Rodríguez-Palomares Jose, Ruiz-Muñoz Aroa, Dux-Santoy Lydia, Villalva Nicolas, Granato Chiara, Galian Laura, Gutiérrez Laura, González-Alujas Teresa, Sanchez Violeta, Forteza Alberto, García-Dorado David, Evangelista Artur (2019). Proximal aorta longitudinal strain predicts aortic root dilation rate and aortic events in Marfan syndrome. European Heart Journal.

[23] Caro CG, Doorly DJ, Tarnawski M, Scott KT, Long Q, Dumoulin CL (1996). Non-planar curvature and branching of arteries and non-planar-type flow. Proc R Soc A Math Phys Eng Sci.

[24] Stonebridge PA, Hoskins PR, Allan PL, Belch JFF (1996). Spiral laminar flow in vivo. Clin Sci.

[25] Morbiducci U, Ponzini R, Rizzo G (2011). Mechanistic insight into the physiological relevance of helical blood flow in the human aorta: an in vivo study. Biomech Model Mechanobiol.

[26] Xiao L, Sun A, Fan Y, Deng X (2015). Physiological significance of helical flow in the arterial system and its potential clinical applications. Ann Biomed Eng.

[27] Zhan F, Fan Y, Deng X (2010). Swirling flow created in a glass tube suppressed platelet adhesion to the surface of the tube : its implication in the design of small-caliber arterial grafts. Thromb Res.

[28] Morbiducci U, Ponzini R, Rizzo G (2009). In vivo quantification of helical blood flow in human aorta by time-resolved three-dimensional cine phase contrast magnetic resonance imaging. Ann Biomed Eng.

[29] Stonebridge PA, Brophy CM (1991). Spiral laminar flow in arteries ? Erythropoietin and spontaneous platelet aggregation in haemodialysis patients. Lancet.

[30] Bissell MM, Hess AT, Biasiolli L (2013). Aortic dilation in bicuspid aortic valve disease: flow pattern is a major contributor and differs with valve fusion type. Circ Cardiovasc Imaging.

[31] Sailer AM, Wagemans BAJM, Das M (2015). Quantification of respiratory movement of the aorta and side branches. J Endovasc Ther.

[32] Claessen BEPM, van der Schaaf RJ, Verouden NJ (2009). Evaluation of the effect of a concurrent chronic Total occlusion on Long-term mortality and left ventricular function in patients after primary percutaneous coronary intervention. JACC Cardiovasc Interv.

