# Supplementary material for: Decreased rotational flow and circumferential wall shear stress as early markers of descending aorta dilation in Marfan syndrome: a 4D flow CMR study
Source: J Cardiovasc Magn Reson. 2019 Oct 14;21:63. doi: 10.1186/s12968-019-0572-1 (PMC6791020; doi:10.1186/s12968-019-0572-1)
Supplement: Supplementary file 2 — Numerical results included in Fig. 2.(DOCX 27 kb) [file 12968_2019_572_MOESM1_ESM.docx]

# Additional file

|  | plane: | HV | ALL MFS | |  | non-dilated MFN | |  |
| --- | --- | --- | --- | --- | --- | --- | --- | --- |
|  |  |  |  | univariate | multivariate |  | univariate | multivariate |
|  |  |  |  | p-values | p-values |  | p-values | p-values |
| IRF  [cm^2^/s] | 1 | -1.77±16.76 | 9.96±36.47 | 0.055 | NS | -0.56±24.15 | 0.823 |  |
|  | 2 | -0.54±25.21 | 7.48±38.82 | 0.239 |  | -5.41±19.77 | 0.461 |  |
|  | 3 | 2.32±31.12 | 1.37±23.80 | 0.855 |  | -1.84±18.15 | 0.591 |  |
|  | 4 | 8.69±33.92 | 3.22±18.90 | 0.275 |  | 0.38±19.76 | 0.327 |  |
|  | 5 | 18.41±35.20 | 9.50±18.95 | 0.084 | 0.025 | 1.18±13.58 | 0.044 | 0.024 |
|  | 6 | 23.52±32.91 | 14.00±20.75 | 0.063 | NS | 9.06±18.54 | 0.080 | NS |
|  | 7 | 30.16±33.74 | 14.21±17.13 | 0.001 | 0.007 | 10.49±16.15 | 0.019 | NS |
|  | 8 | 29.41±36.54 | 12.73±16.77 | 0.001 | 0.048 | 11.17±11.56 | 0.038 | NS |
|  | 9 | 29.64±34.63 | 14.69±19.83 | 0.004 | NS | 14.84±21.26 | 0.092 | NS |
|  | 10 | 29.99±36.22 | 16.01±19.00 | 0.008 | NS | 17.87±20.44 | 0.180 | NS |
|  | 11 | 28.12±34.28 | 12.71±17.62 | 0.002 | 0.050 | 13.47±15.49 | 0.081 | NS |
|  | 12 | 25.60±24.10 | 14.00±15.08 | 0.002 | 0.004 | 15.36±13.66 | 0.090 | NS |
|  | 13 | 28.36±26.78 | 12.16±16.32 | <0.001 | 0.003 | 16.07±15.59 | 0.069 | 0.034 |
|  | 14 | 28.26±27.61 | 10.43±16.10 | <0.001 | 0.003 | 14.17±14.56 | 0.041 | NS |
|  | 15 | 23.71±25.84 | 7.26±18.45 | <0.001 | 0.049 | 11.32±10.49 | 0.049 | 0.050 |
|  | 16 | 22.47±21.43 | 5.20±18.39 | <0.001 | 0.003 | 8.81±14.29 | 0.014 | 0.027 |
|  | 17 | 15.50±23.18 | -4.08±20.23 | <0.001 | 0.023 | 0.61±23.53 | 0.025 | 0.020 |
|  | 18 | 8.16±21.81 | -8.18±20.62 | <0.001 | NS | -0.82±13.19 | 0.103 | 0.017 |
|  | 19 | 2.43±21.29 | -8.83±17.84 | 0.003 | NS | -0.40±14.58 | 0.603 |  |
|  | 20 | -0.17±16.93 | -6.35±15.34 | 0.049 | NS | -2.40±13.46 | 0.616 |  |
| SFRR  [%] | 1 | 5.92±5.46 | 9.04±8.69 | 0.041 | NS | 4.55±3.94 | 0.331 |  |
|  | 2 | 6.68±6.08 | 9.38±8.36 | 0.073 | NS | 5.42±4.26 | 0.421 |  |
|  | 3 | 7.37±7.14 | 8.37±7.39 | 0.488 |  | 5.69±4.12 | 0.345 |  |
|  | 4 | 7.03±5.96 | 7.12±7.93 | 0.951 |  | 4.43±3.52 | 0.083 | NS |
|  | 5 | 6.83±5.35 | 6.06±7.83 | 0.574 |  | 4.30±3.24 | 0.062 | NS |
|  | 6 | 6.77±5.55 | 5.83±7.83 | 0.502 |  | 4.26±3.29 | 0.073 | NS |
|  | 7 | 7.13±6.77 | 6.55±11.73 | 0.772 |  | 4.76±4.40 | 0.169 |  |
|  | 8 | 7.50±7.00 | 6.43±9.33 | 0.527 |  | 5.23±5.01 | 0.210 |  |
|  | 9 | 7.87±7.77 | 7.57±9.64 | 0.867 |  | 6.45±7.94 | 0.514 |  |
|  | 10 | 7.59±6.37 | 7.14±7.24 | 0.744 |  | 6.24±4.94 | 0.421 |  |
|  | 11 | 6.86±5.59 | 6.80±6.50 | 0.962 |  | 6.11±5.57 | 0.629 |  |
|  | 12 | 5.35±4.38 | 5.82±4.97 | 0.617 |  | 4.96±3.51 | 0.732 |  |
|  | 13 | 5.34±4.48 | 6.01±4.91 | 0.478 |  | 4.83±4.37 | 0.677 |  |
|  | 14 | 4.93±5.05 | 4.95±3.64 | 0.975 |  | 4.74±3.08 | 0.883 |  |
|  | 15 | 4.35±3.44 | 4.54±4.19 | 0.804 |  | 4.00±3.06 | 0.709 |  |
|  | 16 | 3.76±3.23 | 4.79±4.03 | 0.165 |  | 3.90±2.86 | 0.867 |  |
|  | 17 | 3.92±3.83 | 5.01±4.20 | 0.174 |  | 3.06±2.20 | 0.369 |  |
|  | 18 | 3.26±2.85 | 4.28±4.34 | 0.181 |  | 2.54±1.47 | 0.301 |  |
|  | 19 | 2.94±2.49 | 3.84±4.35 | 0.226 |  | 2.37±1.48 | 0.365 |  |
|  | 20 | 2.61±2.66 | 3.62±3.96 | 0.149 | NS | 2.43±1.75 | 0.784 |  |

Table S1. In-plane rotational flow (IRF) (top) and systolic flow reversal ratio (SFRR) (bottom) at the 20 analysis planes in healthy volunteers (HV), all Marfan patients (ALL MFS) and non-dilated Marfan patients. NS = non-significant.
